# M(VI) Oxidation State Stabilization in Iron, Cobalt and Nickel Heteroligand Metal Chelates Containing 3,7,11,15-Tetraazaporphine and Two Axial Oxo Ligands: Quantum-Chemical Simulation

**DOI:** 10.3390/ijms21041494

**Published:** 2020-02-22

**Authors:** Oleg V. Mikhailov, Denis V. Chachkov

**Affiliations:** 1 Analytical Chemistry, Certification and Quality Management, Kazan National Research Technological University, K. Marx Street 68, 420015 Kazan, Russia; 2 Kazan Department of Joint Supercomputer Center of Russian Academy of Sciences – Branch of Federal Scientific Center “Scientific Research Institute for System Analysis of the RAS”, Lobachevskii Street 2/31, 420111 Kazan, Russia; de2005c@gmail.com

**Keywords:** iron(VI): cobalt(VI), nickel(VI), oxo ligand, 3,7,11,15-tetraazaporphine, DFT method

## Abstract

The quantum-chemical calculation of iron, cobalt and nickel heteroligand complexes with the double deprotonated form of (NNNN)-donor atomic ligand—3,7,11,15-tetraazaporphine—and two oxo ligands has been carried out. Data on the structural and standard thermodynamic parameters, NBO analysis and multiplicity of the ground states of these complexes have been presented. The given calculation has been made by using the density functional theory (DFT) method with the OPBE/TZVP basis set. Based on the results of this calculation, the possibility of the existence of oxidation state VI for the chemical elements indicated above—unusual for iron and cobalt, and for nickel, unknown at all—has been shown.

## 1. Introduction

As known, the maximal oxidation state reliably found for iron is VIII [1,2,3,4] and for cobalt and nickel is IV [1,5]. These high-oxidation states were found for oxo compounds FeO_4_, CoO_2_ and NiO_2_ and also for fluorine compounds Cs_2_[CoF_6_], NiF_4_ and K_2_[NiF_6_]. In the literature, there are separate indications on the possibility of the existence of cobalt and nickel compounds with higher oxidation states than IV, but there is no specific information on this subject (both experimentally and theoretically). Besides, as follows from the data [1,2,3,4,5], the stabilization of the above-mentioned oxidation states of iron, cobalt and nickel was carried out due to the presence of atoms of one of the two elements with the highest electronegativity, namely oxygen and fluorine. However, it is well known that stabilization of oxidation states uncharacteristic for any particular *d*-element can be achieved using complexing processes with certain specific polydentate (and, in particular, macrocyclic) ligands. Among these ligands, macrocyclic organic compounds containing four donor nitrogen atoms, each of which is capable of forming a chemical bond with different atoms of *p*-, *d*- and *f*-elements, are very promising for solving this problem. As a result, very stable metal complexes with closed contour and four articulated metal chelate rings are formed. According to numerous data, in particular [6,7,8,9,10], such ligands include porphyrin, 3,7,11,15-tetraazaporphine (porphyrazine) and their alkyl, aryl, and halogen derivatives, capable of stabilizing, in principle, both abnormally-high and abnormally-low oxidation states of various *d*-elements. In our previous works [11,12,13], using the quantum-chemical calculation by the density functional theory (DFT) method with the OPBE/TZVP basis set, we showed the possibility of the existence of zinc and copper complexes in oxidation states unusual for them; namely, Zn(III) and Cu(IV), in the inner sphere of which are such tetradentate (NNNN) macrocyclic ligands as 3,7,11,15-tetraazaporphine (porphyrazine) and phthalocyanine, in combination with one or two monodentate ligands having donor atoms with high electronegativity, namely F^–^. In this connection, it seems appropriate to establish whether some of the above macrocyclic ligands can stabilize even higher oxidation states than IV, in particular VI, for such 3*d*-elements as iron, cobalt and nickel. As another ligand having atoms with high electronegativity, an oxo anion O^2–^ may be used. In such a case, as is easy to see, for the realization of such an oxidation state, two oxo anions must also be included in the metal complex composition along with the above macrocyclic ligand. However, there is no information on this subject in the literature, and in this paper we will discuss this possibility for complexes of the given 3*d*-elements with one of the simplest macrocyclic ligands of this type—3,7,11,15-tetraazaporphine (H_2_**L**)—and two O^2–^ anions having [M**L**(O)_2_] composition, with the general formula I (where M = Fe, Co, Ni, and **L** is the double deprotonated form of macrocyclic

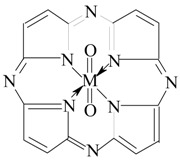
(I)
ligand indicated above), using for this purpose a quantum-chemical calculation by the density functional method.

## 2. Results

**Figure 1:**
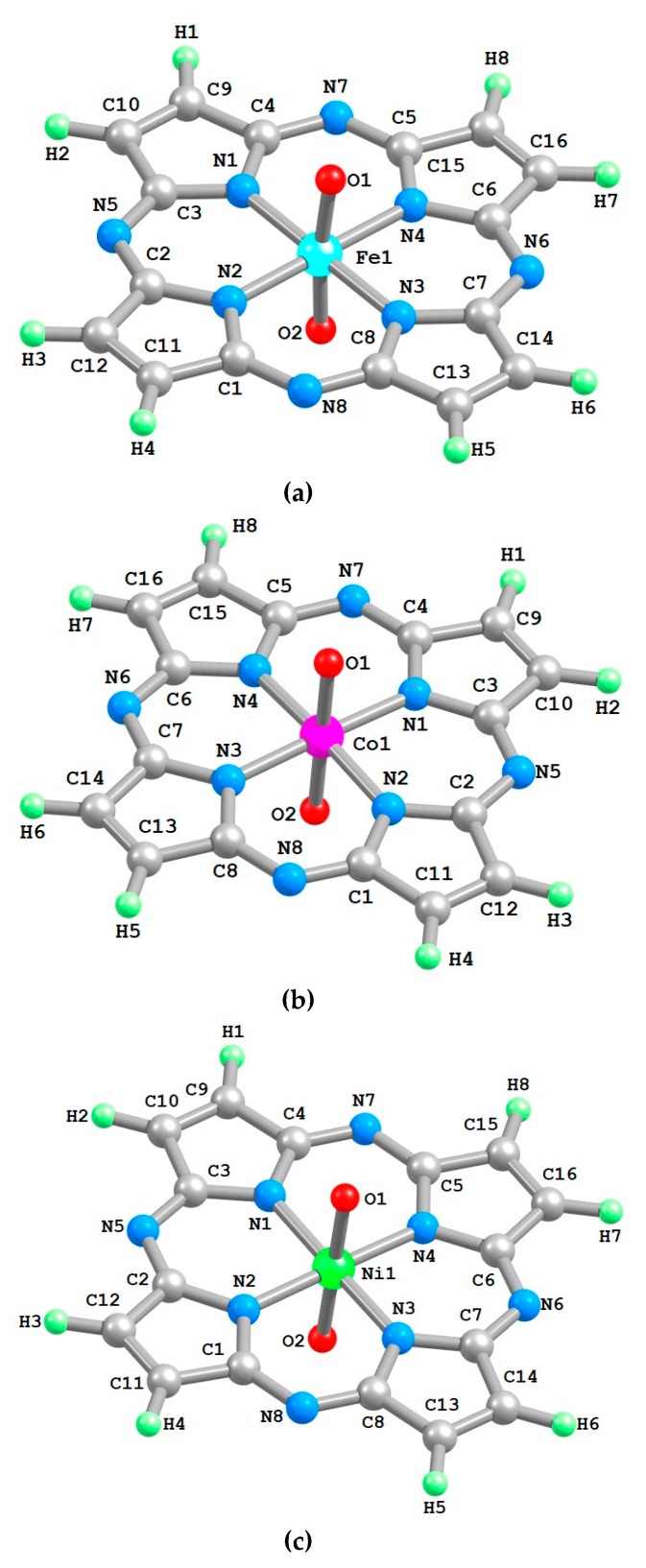
Molecular structures of [M**L**(O)_2_] complexes obtained as a result of the density functional theory (DFT) OPBE/TZVP method quantum-chemical calculation: **a**: Fe(VI), **b**: Co(VI), **c**: Ni(VI).

The calculated chemical bond lengths between atoms and bond angles for this compound are presented in Table 1. Molecular structures of these complexes are shown in Figure 1.

The values of the dipole electric moments for this complex, calculated using the DFT OPBE/TZVP method, are 0.14 ([Fe**L**(O)_2_), 0.02 ([Co**L**(O)_2_) and 0.00 ([Ni**L**(O)_2_) Debye units.

The standard thermodynamic parameters of formation (*ΔH*^0^*_f_*_, 298_, *S*^0^*_f_*_, 298_ and *ΔG*^0^*_f_*_, 298_) for the given macrocyclic metal chelates under examination are presented in Table 2.

NBO analysis data (see Supplementary Materials) show that in the complexes under examination, a very high degree of electron density delocalization occurs (the charge on the M atom is –0.1547 ē (in the iron complex), 0.0647 ē (in the case of cobalt) and 0.3015 ē (in the case of nickel). At the same time, the charges on the oxygen atoms are –0.0178 ē, –0.0895 ē and –0.1636 ē, respectively. The values of <S**2> are 0.000 (Fe), 0.7609 (Co) and 2.0122 (Ni). The ground states of the M(VI) heteroligand complexes of Type I are, in the framework of the DFT OPBE/TZVP method, a spin singlet in the case of [Fe**L**(O)_2_], a spin doublet in the case of [Co**L**(O)_2_] and a spin triplet in the case of [Ni**L**(O)_2_]. Testing the wave functions of the ground state for stability within each of these methods using the STABLE = OPT procedure shows that the wave function of ground state for M_S_ = 1 ([Fe**L**(O)_2_]), 2 ([Co**L**(O)_2_]) and 3 ([Ni**L**(O)_2_]) is stable under the perturbations considered, whereas the wave function of ground states for M_S_ = 3, 4 and 1, respectively, have an internal instability.

## 3. Discussion

From the data in Table 1, it can be seen that both chelate node MN_4_ and all four six-membered metal chelate rings and, also, all four five-membered nonchelate rings containing one nitrogen atom and four carbon atoms and adjacent to six-membered metal chelate rings in the metal macrocyclic compounds under study, have either strong planar structure or practically planar structure. Similar conclusions can be made if we take into consideration the sum of the bond angles in each of these structural fragments (360.0°, 720.0° and 540.0°, respectively). It should be noted that, despite the same bond angles sum (BAS) values, there are certain differences in the sets of valence angles (MNM) in the chelate nodes of these complexes: in the [Ni**L**(O)_2_] complex, all these angles are the same and equal to 90°, whereas in the [Co**L**(O)_2_] complex, they are equal only in pairs, and in the [Fe**L**(O)_2_] complex, only two of the four angles are equal to each other, while the rest are different from each other. It is interesting that in the case of nonvalence angles in the N_4_ groupings forming chelate sites, for the [Fe**L**(O)_2_] and [Co**L**(O)_2_] complexes, the situation is opposite to that observed in the case of valence angles (MNM): pairwise equality of angles occurs in the iron complex; in the cobalt complex, three angles of four are not equal to each other. Accordingly, in the complexes [Fe**L**(O)_2_] and [Co**L**(O)_2_], the sets of angles in metal chelate rings turn out to be somewhat different (although the sums of these angles in each of these complexes are the same, namely 720° in any of these rings) (Table 1). At the same time, the five-membered cycles in each of these complexes are exactly the same both in the total sum of the angles and in their sets. The oxygen and M atoms in the complexes under study form between themselves angles nearly to 180° (in the case of iron and cobalt complexes) and equal to 180° (in the case of the nickel complex). It is noteworthy that, although the chelate node MN_4_ in each of these complexes is strictly flat, the (OMN) bond angles formed by M atoms, donor nitrogen atoms and oxygen atoms are not equal to 90°. Moreover, they differ quite noticeably between the particular one, which is very well manifested in the [Fe**L**(O)_2_] and [Co**L**(O)_2_] complexes. Despite this, these bond angles formed by the same nitrogen donor atom but different oxygen atoms are equal to each other (for example, in the [Ni**L**(O)_2_] complex, (O1M1N2) = (O2M1N2) = 90.8°). Besides, bond lengths between M and N atoms are significantly longer than bond lengths between M and O atoms (Table 1); their lengths correspond to lengths of single M–N and double M=O bonds, respectively. These noted facts, as well as the fact that bond lengths indicated above are different among themselves, make it possible to assign the compounds of Type I under study to the number of pseudo-octahedral complexes with tetragonal distortion. It should be noted in this connection that the values of the dipole electric moments for these complexes calculated using above-mentioned DFT are near zero ([Fe**L**(O)_2_], [Co**L**(O)_2_]) or even equal to 0.00 Debye units ([Ni**L**(O)_2_]), which is in full accordance with the strong planar structure of the last complex with a center of symmetry.

All of the standard thermodynamic parameters of formation (*ΔH*^0^*_f_*_, 298_, *S*^0^*_f_*_, 298_ and *ΔG*^0^*_f_*_, 298_) for the heteroligand complexes under study, as may be seen in Table 2, are positive. Hence, none of them can be obtained from the simple substances formed by chemical elements in their compositions (i.e., C, N, O and Fe, Co, Ni). Nevertheless, according to data obtained as a result of the quantum-chemical calculation, the molecular structures of Type I compounds and the full totality of their geometric parameters are such that each of them can be realized as a single whole. Thus, it can be argued that the complexes of Type I are capable of self-existence, at least in the gas phase. If so, then, in accordance with the generally accepted definition of the term “oxidation degree”—namely, “the oxidation degree is the charge in units of electron charge that would occur on the atom of a given element in a given chemical compound under the assumption that within the framework of each of the conditionally-existing, in this compound, two-center two-electron chemical bonds formed by the exchange mechanism, there would be a complete transfer of electrons from the atom with less electronegativity to the atom with more electronegativity”—the oxidation degree of any of the central atoms considered here (Fe, Co, Ni) is +6 because in any of these compounds, six bonds are formed by these atoms with atoms with greater electronegativity on the exchange mechanism: two with nitrogen atoms and four with oxygen atoms. Accordingly, the oxidation state of each of the chemical elements indicated above (which, as known, if it has a “+” sign, is actually a module of oxidation degree expressed in the corresponding Roman numeral), in each of the [M**L**(O)_2_] compounds is VI. Also, the real charge on the M atoms considered in this article differs significantly from +6.00, but this parameter does not fall within the above definition; therefore, in principle, it cannot be considered an actual oxidation state.

As indicated above, the [Ni**L**(O)_2_] complex has M_S_ = 3; hence, is a low-spin complex. Such a situation is quite expected for pseudo-octahedral Ni(VI) complexes with 3*d*^4^ configuration and a coordination number of a metal ion equal to 6. However, for [Fe**L**(O)_2_] and [Co**L**(O)_2_] where M_S_ = 1 and 2, that is somewhat unexpected for Fe(VI) and Co(VI) complexes with a similar structure having 3*d*^2^ and 3*d*^3^ configurations, where one would expect, as the ground states, the spin triplet and spin quartet, respectively. In our opinion, this circumstance serves as a clear indication of the high degree of delocalization of electron density in these complexes, as a result of which the classical model of the e-ligand field theory—in which it is postulated that the 3*d* electrons M can be on *t_2g_* and *e_g_* MO—does not work anymore. This conclusion is also supported by the NBO analysis data, according to which the effective charges on M atoms in each of the [M**L**(O)_2_] complexes under consideration are extremely low, and, on the contrary, are unusually high on oxygen atoms (see Supplementary Materials). It should also be noted in this connection that, according to the data of our calculations, the nearest excited states of these complexes (triplet in the case of [Fe**L**(O)_2_], quartet in the case of [Co**L**(O)_2_] and singlet in the case of [Ni**L**(O)_2_]) have energies 53.2, 64.2 and 105.9 kJ/mol higher, respectively, than the energies of the ground states of these complexes. This circumstance makes the availability of a spin crossover for it impossible.

## 4. Materials and Methods

As in earlier articles [11,12,13], quantum-chemical calculations were performed by the DFT method with the OPBE/TZVP basis set combined with the common TZVP extended triple-zeta split-valence basis set [14,15] and the OPBE nonhybrid functional [16,17]. As shown in [17,18,19,20,21], in the case of 3*d-*elements, it more adequately predicts the relative energy stabilities of high-spin and low-spin states and reliably characterizes key geometric parameters of corresponding molecular structures. This conclusion is in full harmony with the data of structural parameters of macrocyclic complexes of various 3*d*-elements with phthalocyanine obtained as a result of various DFT quantum-chemical calculations and in experiments (see Supplementary Materials). Calculations were performed with the Gaussian09 program package [22]. The correspondence of the found stationary points to energy minima was proved in all cases by the calculation of second derivatives of energy with respect to atom coordinates; all equilibrium structures corresponding to minima of the potential energy surfaces had only real positive frequency values. Theoretically, Fe(VI) must have 3*d*^2^ electronic configuration. Ni(VI) must have 3*d*^4^ and, in this connection, spin multiplicities 1 and 3 were considered in the calculation. Co(VI) must have 3*d*^3^ electronic configuration, and spin multiplicities 2 and 4 were considered for it. Among the structures optimized at these multiplicities, the lowest-lying structure was selected. Parameters of molecular structures with the given multiplicities were always calculated by the unrestricted (UOPBE) method (for M_S_ = 2, 3 and 4) as well as the restricted (ROPBE) one (for M_S_ = 1). At spin multiplicity 1, the unrestricted method in combination with the option GUESS = Mix was also used; the results thus obtained were similar to those obtained by the restricted method. Also, testing the wave functions of the ground state for stability using the STABLE = OPT procedure was carried out. The standard thermodynamic parameters of formation (*ΔH*^0^*_f_*_, 298_, *S*^0^*_f_*_, 298_ and *ΔG*^0^*_f_*_, 298_) for the given macrocyclic metal chelates under examination were calculated using the method described in [23].

It should be noted especially that the full guarantee that during our calculations we found namely the global minimum and not a local minimum is only a complete calculation of the potential energy surface (PES) for each of metal complexes under examination. However, for such a number of atoms, as takes place in our complexes, the complete calculation of PES is not technically feasible. In this regard, we, like most other researchers (see, e.g., articles [24,25] where *d*-element compounds having oxidation states IX and X were described), work only with a certain PES site. We are looking for local minima in the studied area of the potential energy surface and distinguish the global one among them. Moreover, we note that no other stable structures and conformations for the complexes considered in the article are found according to the data of our calculation. Nevertheless, a minimum found by us for each of the complexes studied is not an artifact that is indirectly confirmed by the stability of the corresponding wave functions of the given complexes.

## 5. Conclusions

As can be seen from the above data, the DFT method used by us, namely the OPBE/TZVP basis set, unambiguously predicts the possibility of the existence of Fe(VI), Co(VI) and Ni(VI) complexes having [M**L**(O)_2_] general formulae where **L** is double deprotonated form 3,7,11,15-tetraazaporphine. The direction for future work is to find each of them in experiments.

## Figures and Tables

**Table 1:** Bond lengths and bond angles in the [M**L**(O)_2_] complexes of Type I (M = Fe, Co, Ni).

Structural Parameter	[FeL(O)_2_]	[CoL(O)_2_]	[NiL(O)_2_]
M–N bond lengths in chelate node MN_4_, *pm*			
M1N1	193.8	192.2	191.6
M1N2	193.8	193.2	191.6
M1N3	196.3	194.2	191.6
M1N4	196.3	193.2	191.6
C–N bond lengths in 6-numbered chelate rings, *pm*			
N1C3	136.3	136.4	136.5
N1C4	136.4	136.4	136.5
N2C1	136.4	136.3	136.5
N2C2	136.3	136.2	136.5
N3C7	136.2	136.7	136.5
N3C8	136.1	136.7	136.5
N4C5	136.1	136.2	136.5
N4C6	136.2	136.3	136.5
N5C2	132.7	132.4	132.1
N5C3	132.7	132.4	132.1
N6C6	132.7	132.4	132.1
N6C7	132.7	132.3	132.1
N7C4	132.7	132.4	132.1
N7C5	132.7	132.4	132.1
N8C1	132.7	132.4	132.1
N8C8	132.7	132.3	132.1
C–C bond lengths in 5-numbered chelate ring (C1N1C4C9C10C3), *pm*			
C4C9	145.3	145.2	145.0
C9C10	135.8	135.7	135.6
C10C3	145.3	145.2	145.0
M–O bond lengths, *pm*			
M1O1	162.7	168.9	180.8
M1O2	162.7	168.9	180.8
Bond angles in chelate node MN_4_, *deg*			
(N1M1N2)	90.4	90.1	90.0
(N2M1N3)	90.0	89.9	90.0
(N3M1N4)	89.6	89.9	90.0
(N4M1N1)	90.0	90.1	90.0
Bond angles sum (*BAS*), *deg*	360.0	360.0	360.0
Non-bond angles between N atoms in N_4_ grouping, *deg*			
(N1N2N3)	90.2	90.0	90.0
(N2N3N4)	89.8	89.8	90.0
(N3N4N1)	89.8	90.0	90.0
(N4N1N2)	90.2	90.2	90.0
Non-bond angles sum (NBAS), *deg*	360.0	360.0	360.0
Bond angles in 6-numbered chelate ring (M1N1C4N7C5N4), *deg*			
(M1N1C4)	125.9	126.1	126.2
(N1C4N7)	128.4	128.3	128.5
(C4N7C5)	122.1	121.3	120.6
(N7C5N4)	127.9	128.1	128.5
(C5N4M1)	125.7	126.1	126.2
(N4M1N1)	90.0	90.1	90.0
Bond angles sum (BAS^61^), *deg*	720.0	720.0	720.0
Bond angles in 6-numbered chelate ring (M1N2C2N5C3N1), *deg*			
(M1N2C2)	125.8	126.1	126.2
(N2C2N5)	128.1	128.1	128.5
(C2N5C3)	121.9	121.3	120.6
(N5C3N1)	128.1	128.3	128.5
(C3N1M1)	125.7	126.1	126.2
(N1M1N2)	90.4	90.1	90.0
Bond angles sum (BAS^62^), *deg*	720.0	720.0	720.0
Bond angles in 6-numbered chelate ring (M1N3C8N8C1N2), *deg*			
(M1N3C8)	125.7	125.9	126.2
(N3C8N8)	127.9	128.2	128.5
(C8N8C1)	122.1	121.4	120.6
(N8C1N2)	128.4	128.5	128.5
(C1N2M1)	125.9	126.1	126.2
(N2M1N3)	90.0	89.9	90.0
Bond angles sum (BAS^63^), *deg*	720.0	720.0	720.0
Bond angles in 6-numbered chelate ring (M1N4C6N6C7N3), *deg*			
(M1N4C6)	125.9	126.1	126.2
(N4C6N6)	128.2	128.5	128.5
(C6N6C7)	122.2	121.4	120.6
(N6C7N3)	128.2	128.2	128.5
(C7N3M1)	125.9	125.9	126.2
(N3M1N4)	89.6	89.9	90.0
Bond angles sum (BAS^64^), *deg*	720.0	720.0	720.0
Bond angles in 5-numbered ring (C3N1C4C9C10), *deg*			
(C3N1C4)	108.3	107.8	107.4
(N1C4C9)	108.7	109.1	109.3
(C4C9C10)	107.1	107.0	107.0
(C9C10C3)	107.1	107.0	107.0
(C10C3N1)	108.8	109.1	109.1
Bond angles sum (BAS^51^), *deg*	540.0	540.0	540.0
Bond angles between O, M and N atoms, *deg*			
O1M1N1	92.4	94.9	89.2
O1M1N2	92.5	90.0	90.8
O1M1N3	87.6	85.1	89.2
O1M1N4	87.5	90.0	90.8
Bond angles between O, M and O atoms, *deg*			
O1M1O2	173.1	170.1	180.0

**Table 2:** Standard thermodynamic parameters of formation (*ΔH*^0^*_f_*_, 298_, *S*^0^*_f_*_, 298_ and *ΔG*^0^*_f_*_, 298_) for the complexes of Type I.

Complex	*ΔH*^0^*_f_*_, 298_, kJ/mole	*S*^0^*_f_*_, 298_, J/mole ∙K	*ΔG*^0^*_f_*_, 298_, kJ/mole
[FeL(O)_2_]	438.2	758.7	663.4
[CoL(O)_2_]	616.3	759.8	842.1
[NiL(O)_2_]	780.9	784.3	999.3

## Supplementary Materials

The following are available online at https://www.mdpi.com/1422-0067/21/4/1494/s1.
